# Clinical impact of treprostinil in neonates with persistent pulmonary hypertension refractory to inhaled nitric oxide: A retrospective cohort study

**DOI:** 10.1097/MD.0000000000046984

**Published:** 2026-01-02

**Authors:** Tae Hyeong Kim, Song Ee Youn, Sung-Hoon Chung

**Affiliations:** a Department of Pediatrics, Kyung Hee University College of Medicine, Kyung Hee University Hospital at Gangdong, Seoul, Korea.

**Keywords:** nitric oxide, oxygenation, persistent pulmonary hypertension of the newborn, premature infant, treprostinil

## Abstract

Persistent pulmonary hypertension of the newborn (PPHN) has limited options when unresponsive to inhaled nitric oxide (iNO). We evaluated the role of intravenous treprostinil and whether early response classification could inform management. We retrospectively analyzed 114 neonates who had received iNO therapy during the period from 2014 to June 2025. Based on oxygenation index change during the 1st 24 hours after iNO initiation, infants were classified as fast, slow, or non-responders. Treprostinil was used selectively in severe cases; among 32 non-responders, 16 received adjunctive intravenous treprostinil starting at 6 ng/kg/min and titrated to a maximum of 20 ng/kg/min by clinical response. Baseline characteristics and outcomes were compared between treprostinil-exposed and unexposed infants in the overall cohort. In the non-responder subgroup, time to taper iNO to 10 ppm was compared and a multivariable Cox model was fitted. Compared with unexposed infants, the treprostinil-exposed group had lower gestational age, higher initial oxygenation index, and more frequent inotropic support, indicating greater illness severity, yet mortality and extracorporeal membrane oxygenation use were not significantly different between the 2 groups. Among non-responders, oxygenation improved more quickly in treprostinil-exposed infants, who achieved iNO tapering to 10 ppm earlier (*P* = .005). Treprostinil exposure was independently associated with earlier clinical stabilization in the Cox model (hazard ratio = 4.99, 95% confidence interval: 1.47–16.98, *P* = .010). No major adverse effects were observed. In neonates with PPHN unresponsive to iNO, intravenous treprostinil was associated with faster improvement in oxygenation and earlier iNO tapering among non-responders, despite greater baseline severity. Classifying early treatment responses could assist in selecting infants who are more likely to benefit from additional therapies.

## 1. Introduction

Persistent pulmonary hypertension of the newborn (PPHN) is a serious condition that can be life-threatening, as it reflects a failure of the normal transition of the circulatory system after birth. When pulmonary vascular resistance remains elevated, blood continues to shunt from right to left through fetal pathways, resulting in marked hypoxemia that requires intensive treatment and monitoring.^[1]^ The incidence of PPHN has been estimated at approximately 1.9 cases per 1000 live births. Even with modern advances in neonatal intensive care, many infants still face serious complications, and mortality remains a concern. This burden is especially evident in preterm infants and in those presenting with profound hypoxemic respiratory failure.^[2]^ At the physiological level, PPHN reflects a combination of impaired vasodilation, structural changes within the pulmonary vasculature, and continued vasoconstriction. Together, these factors prevent the normal postnatal decline in pulmonary arterial pressure.^[3]^ First-line management typically includes mechanical ventilation, optimization of fluid and hemodynamic status, and administration of inhaled nitric oxide (iNO), a selective pulmonary vasodilator that targets cyclic guanosine monophosphate pathways to relax the pulmonary vasculature.^[4]^

However, a significant proportion of affected neonates show either incomplete or absent improvement after exposure to iNO, necessitating the use of alternative or adjunctive strategies.^[5]^ The limited efficacy of iNO may result from multiple mechanisms, including downstream signaling dysfunction, reduced soluble guanylate cyclase activity, and impaired ventilation–perfusion matching.^[6]^ Moreover, the potential toxicity of iNO in underdeveloped lungs, uncertainty surrounding its long-term outcomes, and its limited availability in resource-poor settings point to the necessity of exploring additional or adjunctive therapeutic options.^[4,7]^ Prostacyclin analogs such as treprostinil (Remodulin, United Therapeutics, Silver Spring) have been proposed as potential alternatives. Although not specifically approved for neonatal use, they possess vasodilatory and antiproliferative properties through activation of adenylate cyclase and increased intracellular cyclic adenosine monophosphate, thereby producing pulmonary vasodilation.^[8]^ We previously reported 2 cases of preterm neonates with iNO-refractory PPHN who responded favorably to intravenous treprostinil.^[9]^ Following those initial cases, our center has selectively administered treprostinil to neonates with particularly severe and refractory disease; however, cohort-level evidence regarding its efficacy and safety remains limited.

This study aimed to characterize the patterns of response to iNO in neonates with PPHN and to evaluate the clinical outcomes of adjunctive treprostinil therapy in those who showed little or no response to iNO. We also examined whether gestational age influenced treatment response and overall prognosis. By analyzing a retrospective cohort collected over more than a decade, we sought to clarify the role of treprostinil in neonatal intensive care and to provide evidence to inform management strategies for this high-risk population.

## 2. Methods

### 2.1. Study design and patients

This retrospective, single-center cohort study was conducted at the Neonatal Intensive Care Unit of Kyung Hee University Hospital at Gangdong, Korea, between January 2014 and June 2025. The study was approved by the Institutional Review Board (approval number: KHNMC 2025-08-019), and the requirement for informed consent was waived.

**Figure 1.**
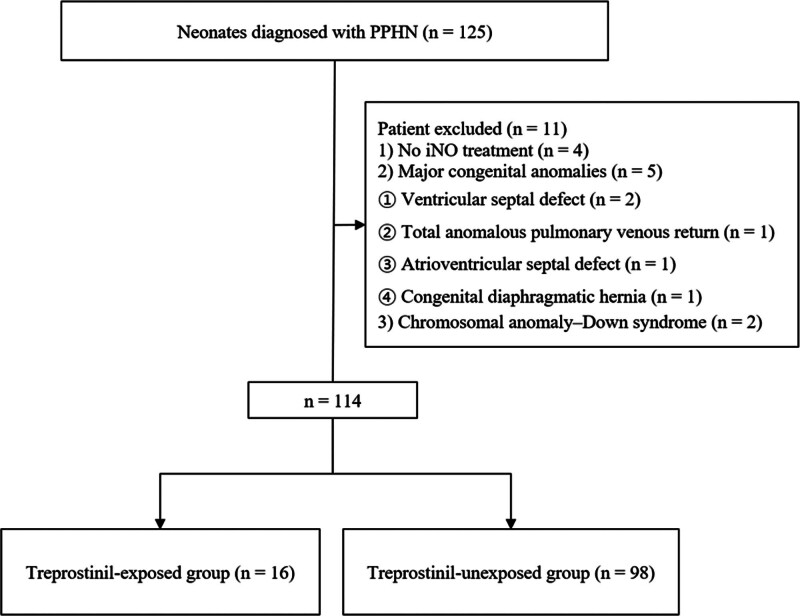
Flowchart of the enrollment of the study participants.

Neonates diagnosed with PPHN who were initiated on iNO therapy within the 1st 2 days of life were included in this study. PPHN was diagnosed based on the clinical signs of hypoxemic respiratory failure accompanied by echocardiographic findings suggestive of elevated pulmonary arterial pressure, including interventricular septal flattening, right-to-left shunting across the patent ductus arteriosus or patent foramen ovale, and tricuspid regurgitation velocity exceeding 3.0 m/s on continuous-wave Doppler, consistent with previous neonatal studies.^[1,6]^ Infants with major congenital heart disease (e.g., total anomalous pulmonary venous return, ventricular septal defect, and atrioventricular septal defect), congenital diaphragmatic hernia, or chromosomal abnormalities including Down syndrome were excluded. Among 125 neonates who were screened, 11 were excluded based on these criteria, and the final cohort consisted of 114 infants (Fig. 1).

### 2.2. Definition of response to iNO and treatment protocols

All patients received standard management, including mechanical ventilation, hemodynamic support, and iNO therapy, according to institutional protocols. The response to iNO was primarily assessed by measuring the changes in the oxygenation index (OI) during the 1st 24 hours. Consistent with prior studies, a ≥20% reduction in the OI was considered a positive response to iNO.^[10–12]^ To further categorize the temporal pattern of the response, patients were classified as fast responders if a ≥20% OI reduction occurred within 6 hours, as slow responders if the same reduction occurred in 6 to 24 hours, and non-responders if OI decreased by < 10% or worsened within 24 hours. This temporal subclassification was developed for the purpose of this study, given the lack of universally established time-based response definitions for neonatal PPHN. In non-responders with severe PPHN, adjunctive intravenous treprostinil was initiated at 6 ng/kg/min and titrated by 2 to 3 ng/kg/min every 4 to 6 hours, depending on the clinical response, oxygenation status, and hemodynamic tolerance. Since June 2016, treprostinil has been considered for infants categorized as iNO non-responders. However, treprostinil administration was not uniformly applied to all non-responders but was selectively initiated based on the overall clinical status, response to supportive care, and physician discretion. Treprostinil is approved for pulmonary arterial hypertension in adults; its use in neonates with PPHN in this study was off-label. Given its off-label use in neonates, we noted possible adverse effects such as hypotension, bleeding tendency, and gastrointestinal problems such as nausea or diarrhea, as described in previous treprostinil studies across different patient populations.^[13–15]^ All infants who received treprostinil were monitored with continuous cardiorespiratory monitoring, including invasive blood pressure monitoring via an arterial line, to enable early detection and prompt management of adverse events.

### 2.3. Data collection

The collected data included maternal characteristics (gestational age, birth weight, sex, Apgar scores, delivery mode, antenatal steroid exposure, pregnancy-induced hypertension, diabetes mellitus, premature rupture of membranes, chorioamnionitis, McDonald cerclage, and small-for-gestational-age status) and neonatal information, including surfactant use, blood culture results, and PPHN etiology. Clinical parameters, such as PPHN diagnosis and iNO initiation timing; partial pressure of oxygen, fraction of inspired oxygen (FiO₂); OI; mean airway pressure (MAP); echocardiographic findings (shunt direction, tricuspid regurgitation velocity, and right ventricular dilatation); use of sedatives, inotropes, and extracorporeal membrane oxygenation (ECMO); ventilation support duration; and treprostinil administration, were recorded. Outcomes included length of hospitalization, peritoneal dialysis, and mortality. Serial oxygenation and hemodynamic parameters (partial pressure of oxygen, FiO₂, OI, and MAP) were measured at baseline and at 6, 12, 24, 36, 48, 72, and 96 hours and 1 week after iNO initiation. The duration of iNO administration was defined as the time from the initiation of therapy (typically starting at 20 ppm) until the concentration was tapered to 10 ppm. Although initial dosing generally began at 20 ppm, titration up to 60 ppm was occasionally performed depending on clinical severity and oxygenation status. For the analysis, the etiologies were grouped into 3 categories: respiratory (including meconium aspiration syndrome and respiratory distress syndrome), perinatal insult (including perinatal asphyxia and sepsis), and idiopathic. Mean blood pressure was not included in the analysis because it fluctuated markedly over time and was influenced by inotrope dosage, making it an unreliable indicator of disease severity. Idiopathic PPHN was defined based on the absence of meconium-stained fluid, clear or mildly hazy lung fields, and poor response to surfactants, reflecting pulmonary circulatory maladaptation without major lung or systemic disease.^[16,17]^

### 2.4. Statistical analysis

All statistical analyses were performed using R version 4.5.1 (R Foundation for Statistical Computing, Vienna, Austria). Continuous variables were expressed as mean ± standard deviation and compared using Student *t* test or the Mann–Whitney *U* test, as appropriate. Categorical variables were compared using the chi-square test or Fisher exact test. Longitudinal changes in OI at predefined time points were assessed to compare trends between groups. Time-to-event curves based on the Kaplan–Meier method were constructed to illustrate the timing of clinically meaningful improvement, defined as tapering of iNO concentration to 10 ppm. Group differences were evaluated using the log-rank test. A Cox proportional hazards model was applied among non-responders to identify factors independently associated with earlier oxygenation improvement, adjusting for sex, gestational age, small-for-gestational-age status, delivery mode, initial OI, 5-minute Apgar score, and PPHN etiology. A 2-sided *P* < .05 was considered statistically significant. All statistical analyses were performed by the corresponding author, who has expertise in statistical analysis of neonatal cohort data.

## 3. Results

A total of 114 neonates with PPHN who were treated with iNO between 2014 and 2025 were included in the final analysis. Based on early changes in the OI, patients were classified into fast, slow, and non-responder groups. Among the 114 neonates, 23 (20.2%) were classified as fast responders, 59 (51.8%) as slow responders, and 32 (28.1%) as non-responders based on changes in the OI within the first 24 hours following iNO initiation.

### 3.1. Baseline characteristics of the treprostinil-exposed and unexposed groups

The mean gestational age of the 114 neonates diagnosed with PPHN was 38.1 ± 3.4 weeks, and 20.2% were born preterm. Most patients were outborn (74.6%), and 51.8% were delivered via cesarean section. The etiologies of PPHN included meconium aspiration syndrome (47.4%), respiratory distress syndrome (23.7%), idiopathic causes (16.7%), and early-onset sepsis (8.8%). Surfactant was administered in 90.4% of the patients. The baseline characteristics of the treprostinil-exposed (n = 16) and unexposed (n = 98) groups are summarized in Table 1. Compared with the unexposed group, preterm birth was more frequent in the treprostinil-exposed group (56.3% vs 14.3%, *P* < .001), along with lower gestational age, birth weight, and Apgar scores at 1 and 5 minutes. Early-onset sepsis was markedly more prevalent in the treprostinil-exposed group (50.0% vs 2.0%, *P* < .001), as were maternal risk factors such as McDonald cerclage and preterm premature rupture of membranes (both *P* < .001). The rates of cesarean section, outborn status, and surfactant use did not differ significantly between the groups.

**Table 1:** Baseline characteristics of the neonates in the treprostinil-exposed and unexposed groups.

Variables	Total (n = 114)	Treprostinil-exposed group (n = 16)	Unexposed group (n = 98)	*P*-value
Gestational age (wk)	38.1 ± 3.4	35.5 ± 4.6	38.5 ± 3.0	.021
Gestational age category (wk)				
<37	23 (20.2)	9 (56.3)	14 (14.3)	<.001
≥37	91 (79.8)	7 (43.8)	84 (85.7)	<.001
Birth weight (g)	2859.7 ± 675.6	2350.0 ± 821.7	2942.9 ± 614.4	.013
Multiple gestation	5 (4.4)	1 (6.3)	4 (4.1)	.537
Small-for-gestational-age status	17 (14.9)	4 (25.0)	13 (13.3)	.255
1-min Apgar score	7.2 ± 2.0	5.6 ± 2.7	7.4 ± 1.8	.029
5-min Apgar score	8.5 ± 1.9	7.0 ± 2.6	8.8 ± 1.7	.016
Cesarean section	59 (51.8)	7 (43.8)	52 (53.1)	.593
Outborn status	85 (74.6)	10 (62.5)	75 (76.5)	.232
Etiology of PPHN				
MAS	54 (47.4)	4 (25.0)	50 (51.0)	.063
RDS	27 (23.7)	1 (6.3)	26 (26.5)	.112
Early-onset sepsis	10 (8.8)	8 (50.0)	2 (2.0)	<.001
Idiopathic causes	19 (16.7)	2 (12.5)	17 (17.3)	1.00
Others	4 (3.5)	1 (6.3)	3 (3.1)	.459
Maternal age	30.5 ± 5.3	32.3 ± 5.2	30.2 ± 5.4	.186
Pregnancy-induced hypertension	28 (24.6)	7 (43.8)	21 (21.4)	.066
Gestational DM	21 (18.4)	5 (31.3)	16 (16.3)	.170
McDonald cerclage	6 (5.3)	5 (31.3)	1 (1.0)	<.001
Surfactant use	103 (90.4)	15 (93.8)	88 (89.8)	1.00
Any PROM type				
Preterm PROM	7 (6.1)	5 (31.3)	2 (2.0)	<.001
Term PROM	14 (12.3)	3 (18.8)	11 (11.2)	.413
Positive blood culture	10 (8.8)	8 (50.0)	2 (2.0)	<.001

Data are presented as mean ± standard deviation for continuous variables and number (%) for categorical variables.

DM = diabetes mellitus, MAS = meconium aspiration syndrome, PPHN = persistent pulmonary hypertension of the newborn, PROM = premature rupture of membranes, RDS = respiratory distress syndrome.

### 3.2. Clinical characteristics and outcomes of the treprostinil-exposed and unexposed groups

The comparative outcomes of the treprostinil-exposed and unexposed neonates are summarized in Table 2. As expected, based on treatment indications, the treprostinil-exposed group included more critically ill infants at baseline. The OI and MAP at the time of iNO initiation were significantly higher in the treprostinil-exposed group than in the unexposed group. Although the unexposed group demonstrated a substantial reduction in the OI at 24 hours (–29.9 ± 39.7%), the treprostinil-exposed group showed a paradoxical increase (+36.1 ± 20.3%, *P* < .001), consistent with their designation as non-responders. The duration of iNO administration was significantly longer in the treprostinil-exposed group than in the unexposed group (121.7 ± 38.1 vs 54.7 ± 25.6 hours, respectively, *P* < .001), as was the duration of mechanical ventilation (12.8 ± 4.3 vs 5.3 ± 3.2 days, respectively, *P* < .001). All infants in the treprostinil-exposed group required inotropic support, compared with 70.4% in the unexposed group (*P* = .011). Peritoneal dialysis was also more frequently required in the treprostinil-exposed group (18.8% vs 1.0% in the unexposed group, *P* = .009), which is in line with the overall higher severity of illness. There were no significant differences in ECMO use or mortality (both 6.3% vs 4.1% in the treprostinil-exposed and unexposed groups, respectively) between the groups. The length of hospitalization was significantly higher in the treprostinil-exposed group than in the unexposed group (50.5 ± 23.7 vs 21.0 ± 13.4 days, respectively, *P* < .001). In addition, no clinically significant adverse events related to treprostinil were observed during treatment. According to the definition used in the study, all infants in the treprostinil-exposed group were classified as non-responders to iNO therapy. Among the unexposed neonates, 23.5% were fast responders, 60.2% were slow responders, and 16.3% were non-responders (*P* < .001).

**Table 2:** Comparative outcomes of the treprostinil-exposed and unexposed neonates.

Variables	Treprostinil-exposed group (n = 16)	Unexposed group (n = 98)	*P*-value
OI at the time of iNO initiation	33.3 ± 4.8	29.6 ± 5.6	.017
MAP at the time of iNO initiation	13.9 ± 1.5	10.8 ± 1.8	<.001
OI at 24 h after iNO initiation	45.0 ± 7.1	21.2 ± 12.5	<.001
Change in the OI at 24 h after iNO initiation (%)	+36.1 ± 20.3	–29.9 ± 39.7	<.001
Duration of iNO administration (h)	121.7 ± 38.1	54.7 ± 25.6	<.001
Duration of mechanical ventilation (d)	12.8 ± 4.3	5.3 ± 3.2	<.001
Inotropics use	16 (100.0)	69 (70.4)	.011
Peritoneal dialysis	3 (18.8)	1 (1.0)	.009
ECMO use	1 (6.3)	4 (4.1)	.537
Mortality before discharge	1 (6.3)	4 (4.1)	.537
Length of hospitalization (d)	50.5 ± 23.7	21.0 ± 13.4	<.001
Responder classification			<.001
Fast responder	0	23 (23.5)	–
Slow responder	0	59 (60.2)	–
Non-responder	16 (100.0)	16 (16.3)	–

Data are presented as mean ± standard deviation or number (percentage).

Duration of iNO administration was defined as the time from the initiation of therapy (typically starting at 20 ppm) until the concentration was tapered to 10 ppm.

ECMO = extracorporeal membrane oxygenation, iNO = inhaled nitric oxide, MAP = mean airway pressure, OI = oxygenation index.

### 3.3. Post-iNO oxygenation trends in non-responders according to treprostinil exposure

**Figure 2.**
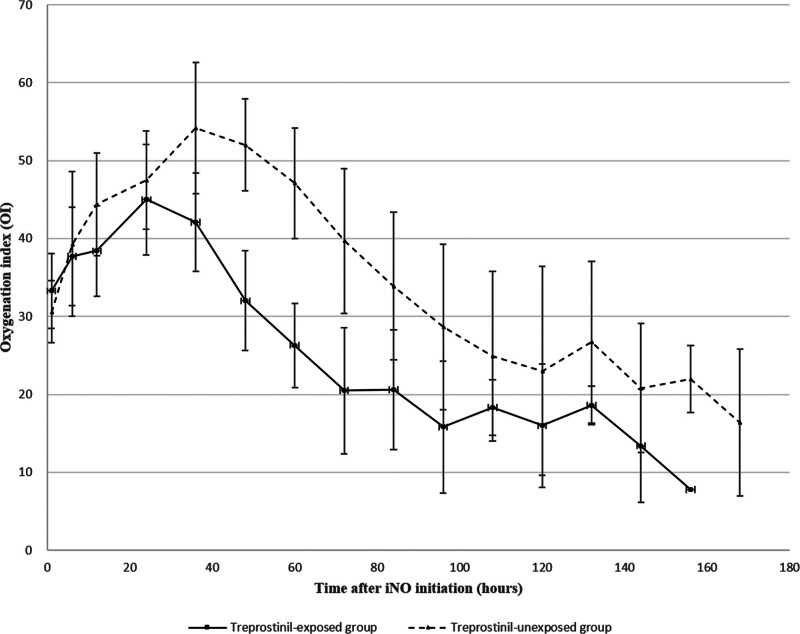
Longitudinal trends in the oxygenation index (OI) over 168 h after iNO initiation in non-responding neonates. Mean ± standard deviation values are shown for each group at serial time points. iNO = inhaled nitric oxide.

**Figure 3.**
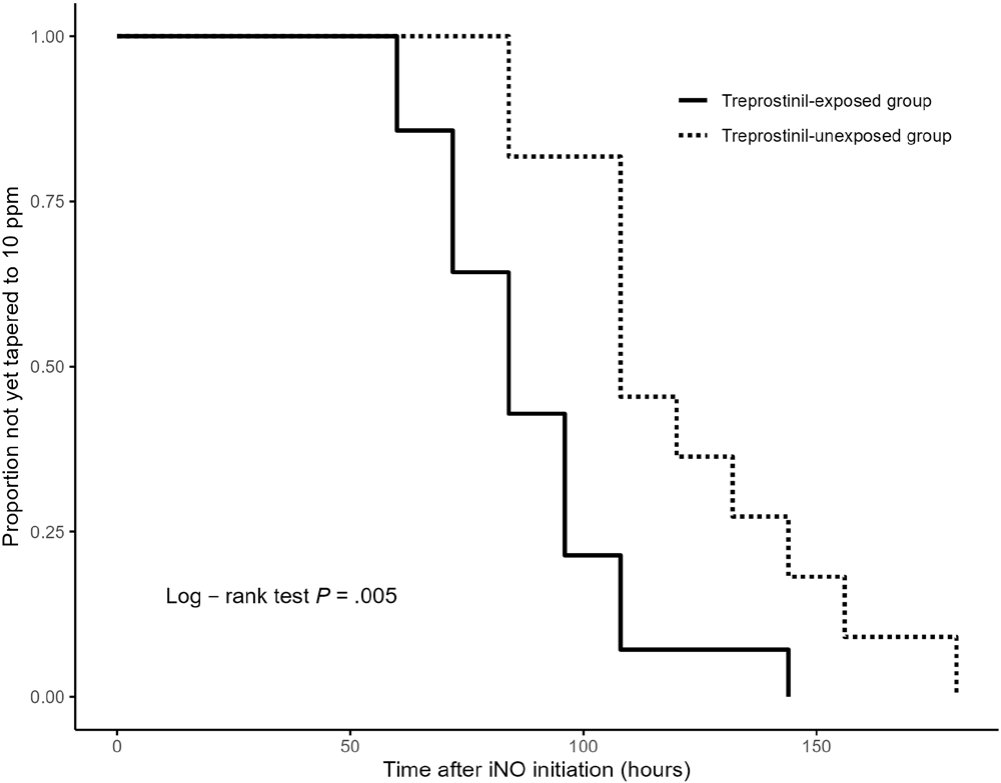
Time-to-event curves for iNO tapering to 10 ppm among non-responders, stratified by treprostinil exposure. The plot shows the proportion of neonates who had not yet reached the point of iNO tapering to 10 ppm over time after initiation. This threshold was used as a surrogate marker of clinical improvement in oxygenation. Solid line represents the treprostinil-exposed group and the dashed line represents the unexposed group. Only neonates classified as iNO non-responders were included in this analysis. Log-rank test *P* = .005. iNO = inhaled nitric oxide.

Figure 2 shows the longitudinal changes in the OI among neonates classified as non-responders to iNO, comparing the treprostinil-exposed and unexposed neonates. In both groups, the OI increased during the 1st 24 hours after iNO initiation. In the treprostinil-exposed group, the OI began to improve at approximately 36 hours, following treprostinil initiation at approximately 24 hours. In contrast, the unexposed group showed a slower and more delayed improvement in oxygenation, with a more gradual decline in the OI over time. Figure 3 presents a time-to-event analysis comparing the duration until iNO was tapered to 10 ppm among non-responders. The treprostinil-exposed group reached this point significantly earlier than the unexposed group did (log-rank test, *P* = .005), suggesting a faster trajectory toward stabilization in infants who received treprostinil.

### 3.4. Factors associated with earlier iNO tapering

A multivariable Cox proportional hazards regression analysis was conducted among the neonates classified as non-responders to identify the factors associated with a shorter duration from iNO initiation until tapering to 10 ppm (Table 3). Treprostinil exposure was the only variable independently associated with earlier iNO tapering (hazard ratio = 4.99, 95% CI: 1.47–16.98, *P* = .010). Other clinical characteristics, including gestational age, sex, small-for-gestational-age status, delivery mode, initial OI, and outborn status, were not significantly related to the outcome. Etiologies, such as idiopathic PPHN, perinatal insult, or respiratory causes, also showed no significant differences in their association with earlier iNO tapering.

**Table 3:** Cox proportional hazards regression identifying the factors associated with earlier iNO tapering in non-responding neonates.

Variables	HR (95% CI)	*P*-value
Treprostinil exposure	4.99 (1.47–16.98)	.010
Gestational age	1.17 (0.94–1.46)	.169
Male sex	0.62 (0.15–2.55)	.508
Small-for-gestational-age status	0.25 (0.02–2.68)	.249
Vaginal delivery	1.71 (0.47–6.31)	.418
Idiopathic etiology (vs respiratory)	2.08 (0.35–12.28)	.418
Perinatal insult (vs respiratory)	1.97 (0.15–25.13)	.602
5-min Apgar score	0.91 (0.58–1.44)	.696
Outborn status	0.44 (0.11–1.84)	.260
Pregnancy-induced hypertension	4.50 (0.93–21.81)	.062
Initial OI	1.11 (0.96–1.29)	.157
Data are presented as HR with 95% CI		

The HRs and 95% CIs were estimated using Cox proportional hazards regression.

The outcome was the time from iNO initiation until tapering to 10 ppm.

An HR > 1 indicates faster achievement of the outcome.

CI = confidence interval, HR = hazard ratio, iNO = inhaled nitric oxide, OI = oxygenation index.

## 4. Discussion

In this retrospective cohort study of 114 neonates with PPHN treated with iNO, we classified patients into fast, slow, and non-responder groups based on early changes in the OI. Non-responders were defined as those whose OI decreased by <10% within the 1st 24 hours after iNO initiation. Approximately 30% of the neonates met the criteria for non-response, while the rest showed either rapid or delayed improvement during this period. Among these non-responders, 50% (16 out of 32 neonates) received adjunctive intravenous treprostinil based on clinical severity and physician discretion. Despite greater baseline illness severity, treprostinil-exposed non-responders showed no increase in ECMO use or mortality compared with unexposed infants. Oxygenation gradually improved in most treprostinil-exposed infants, and they achieved faster tapering of iNO to 10 ppm than the unexposed group. This association was confirmed in the multivariable Cox regression analysis.

These findings are consistent with and extend prior research examining the therapeutic potential of treprostinil in neonatal PPHN. Our observation that treprostinil improved oxygenation in iNO non-responders is in line with Kim et al, who also reported favorable improvements in both term and preterm infants,^[18]^ and with Lawrence et al, who demonstrated improved oxygenation in congenital diaphragmatic hernia-associated PPHN.^[19]^ However, unlike these reports, our study did not show a difference in mortality or ECMO use between treprostinil-treated and untreated infants. This discrepancy may be related to the greater baseline severity of illness in our treprostinil group, as well as the smaller sample size. In addition, while subcutaneous treprostinil has been reported as feasible, including outpatient continuation in infants with chronic lung disease,^[20]^ our cohort was limited to intravenous administration in the acute phase. Regarding safety, no clinically significant adverse events were observed in our cohort, which is consistent with previous neonatal reports. Nonetheless, transient hypotension, tachycardia, or infusion-site complications have been described in other series,^[15,21,22]^ so our negative findings should be interpreted with caution. Overall, our results support the growing evidence that treprostinil provides short-term hemodynamic and oxygenation benefits, while acknowledging ongoing uncertainties regarding survival impact, optimal route of administration, and the need for further studies on long-term outcomes, as emphasized in recent systematic reviews and consensus statements.^[23–25]^

Beyond simple outcome comparisons, this study used early changes in oxygenation to classify the treatment response in a more systematic manner. By focusing on patients who showed minimal improvement within the first 24 hours of iNO therapy, we were able to assess the effects of treprostinil in a group with a clearly limited response to standard care. This targeted approach helped to reduce confounding and provided a clearer picture of how adjunctive therapy might influence the clinical course of more severely affected infants. In this context, the earlier iNO tapering among treprostinil-treated non-responders suggests a possible role of the drug in supporting pulmonary recovery, even in infants with greater illness severity. Although we did not observe significant differences in outcomes, such as ECMO use or mortality, earlier oxygenation improvement may still have meaningful implications for overall clinical stability and resource use. The relationship between gestational age and the treatment response showed a consistent pattern. Preterm infants are more likely to require additional therapy, which may reflect the developmental limitations of pulmonary vascular reactivity.^[26]^ This observation supports the need for more individualized treatment decisions that consider both disease severity and maturity.

During the early phase of treprostinil adoption in our unit, its use was limited by clinical uncertainty and a lack of experience with the drug in neonates. Following the availability of intravenous treprostinil in June 2016, its use in non-responders gradually increased as clinicians gained experience in dosing, monitoring, and short-term responses. However, treatment decisions were based on individual clinical judgments rather than a formal protocol. All patients were initiated at 6 ng/kg/min, with titration in 2 to 3 ng/kg/min increments every 4 to 6 hours, depending on hemodynamic stability. Most infants responded by 20 ng/kg/min, and higher doses were rarely needed. In 1 patient, the dose was increased to 40 ng/kg/min without benefit, and the infant later died of refractory disease. Beyond neonatal data, experience from adult and pediatric populations has reported prostacyclin-class adverse effects such as headache, flushing, diarrhea, and infusion-site pain.^[27–30]^ Although not observed in our cohort, these findings indicate the importance of cautious titration and close monitoring in neonatal practice. Consensus recommendations further emphasize the need for standardized dosing and safety protocols in this population.^[31,32]^

Given the observed association between treprostinil exposure and earlier iNO tapering in non-responders, despite higher illness severity, our findings support its potential role as a rescue therapy in neonates with refractory PPHN, particularly in preterm infants where time-sensitive interventions are critical. Although previous studies, such as a retrospective Chinese cohort study, have reported improved oxygenation and pulmonary pressure with treprostinil compared with oral sildenafil, they often lacked a structured temporal classification or focused primarily on term infants.^[33]^ Similarly, other retrospective studies have described short-term improvements with intravenous treprostinil in both term and preterm neonates but did not apply consistent time-based endpoints or include comparison groups.^[18]^ Unlike earlier studies that primarily described general oxygenation trends, our study incorporated a structured classification of iNO responsiveness based on early OI changes and applied time-to-event analysis to quantify the treatment response. Inhaled iloprost has also been explored as an alternative therapy for neonates with iNO-refractory PPHN. A retrospective review of 22 infants demonstrated significant OI and FiO₂ reductions within 24 hours for nearly half of the cohort, with no serious adverse effects.^[34]^ A larger retrospective study of 51 neonates and infants treated with continuous inhaled iloprost showed decreases in FiO₂ and OI without hemodynamic instability; approximately 47% of the neonates recovered without the requirement of ECMO, although 23% died.^[35]^ Considering these findings, intravenous treprostinil may provide a more consistent pharmacological effect, particularly in situations in which inhaled therapies, including iNO, fail to produce a timely or adequate response. This strategy could be particularly useful in critically ill neonates where achieving early clinical stability is crucial.

This study has some limitations that should be acknowledged. First, this was a retrospective, single-center study, and treprostinil administration was not based on a predefined protocol. Although the study included patients from 2014 onward, treprostinil was only available in our unit in mid-2016. As a result, some early non-responders did not have access to the drug, and subsequent use was determined by individual clinical judgment, which may have introduced a selection bias. Second, the number of treprostinil-exposed infants was small, and events such as ECMO use or death were infrequent, limiting the statistical power of these outcomes. Third, echocardiography was primarily performed at the time of diagnosis and again after clinical improvement; owing to staffing and workload constraints, interim serial assessments were not always feasible. This limited our ability to track detailed hemodynamic changes in relation to treatment. Finally, long-term outcomes, including neurodevelopmental status, were not evaluated.

## 5. Conclusions

Treatment options are limited in neonates with PPHN who do not respond to iNO, and timely clinical decisions are critical. This study suggests that intravenous treprostinil administration may contribute to faster recovery in a subset of these infants, particularly those born preterm. Despite the higher initial illness severity, infants exposed to treprostinil showed more rapid improvement in oxygenation and earlier tapering of iNO compared with their unexposed peers. Although the findings are retrospective and based on a small sample, they offer valuable insights into the potential role of treprostinil as rescue therapy. By using early changes in the OI to classify treatment response, clinicians may be better positioned to tailor management strategies for vulnerable neonates with PPHN. This study emphasizes the importance of individualized treatment approaches and the potential role of treprostinil in promoting clinical stabilization when standard therapies are insufficient.

## Author contributions

**Conceptualization:** Tae Hyeong Kim, Sung-Hoon Chung.

**Data curation:** Tae Hyeong Kim.

**Formal analysis:** Sung-Hoon Chung.

**Investigation:** Tae Hyeong Kim, Song Ee Youn.

**Methodology:** Song Ee Youn.

**Supervision:** Sung-Hoon Chung.

**Validation:** Song Ee Youn.

**Visualization:** Sung-Hoon Chung.

**Writing – original draft:** Tae Hyeong Kim.

**Writing – review & editing:** Song Ee Youn, Sung-Hoon Chung.
